# Utility of extended reality in shoulder arthroplasty: a meta-analysis of training efficiency and glenoid guidewire accuracy

**DOI:** 10.1016/j.jsea.2026.100051

**Published:** 2026-06-24

**Authors:** Max L. Ruiz, Geoffrey D. Abrams, Michael T. Freehill, Emilie V. Cheung

**Affiliations:** Department of Orthopaedic Surgery Stanford Hospitals and Clinics, Stanford, CA, USA

**Keywords:** Extended reality, Virtual reality, Augmented reality, Mixed reality, Shoulder arthroplasty, Surgical education, Glenoid guidewire, Implant positioning

## Abstract

**Background:**

Extended reality (XR) has seen increased usage in shoulder arthroplasty for both intraoperative guidance and surgical training, but its impact on technical accuracy and performance has not been quantified across studies. The objective of this analysis was to determine the impact of XR on educational efficiency and glenoid guidewire placement error. Two separate meta-analyses of randomized or quasi-experimental studies were performed.

**Methods:**

Based on the Preferred Reporting Items for Systematic Reviews and Meta-Analyses search guidelines, 24 studies were identified in XR-based shoulder surgery education. After applying inclusion, 3 studies involving 55 participants were meta-analyzed. Using the same Preferred Reporting Items for Systematic Reviews and Meta-Analyses guidelines, 35 studies were identified in XR-guided shoulder arthroplasty. After applying inclusion criteria, 7 studies involving 349 guidewire placements were meta-analyzed.

**Results:**

In the surgical education studies, XR-trained participants completed procedures faster than controls, but OSAT (Objective Structured Assessment of Technical Skills) scores did not significantly differ. Across the glenoid guidewire studies, XR guidance reduced version error and inclination error versus freehand, but entry point error was not significantly different between XR-guided procedures and freehand.

**Conclusion:**

XR-based tools appear to offer meaningful benefits for shoulder arthroplasty, but their impact may be domain-specific. XR education may substantially improve operative efficiency, while XR guidance may enhance the angular accuracy of glenoid guidewire placement without clearly changing entry point error.

Shoulder arthroplasty is an anatomically complex procedure with a steep learning curve, requiring precise spatial orientation for correct glenoid and humeral component placement.^1^ Shoulder arthroplasty is also associated with a relatively higher complication rate when compared to hip and knee arthroplasty.^21^ Proficiency in component placement is essential for optimal surgical outcomes, yet the variable caseload of shoulder arthroplasty during orthopedic surgery residency may provide limited training opportunities.

Concurrently, surgical education has increasingly embraced competency-based frameworks that utilize objective, scalable assessment tools.^25^ One such framework is Entrustable Professional Activities, a benchmark for assessing orthopedic resident readiness across subspecialties. An Entrustable Professional Activities signifies that a trainee can be trusted to carry out a defined clinical activity without supervision,^29^ and numerous simulation modalities have been developed to bring trainees to this level. Virtual reality (VR), augmented reality (AR), and mixed reality have all gained traction as a means of boosting skill acquisition.^26^^,^^31^ Extended reality (XR) is an umbrella term that is often used to refer to all 3 training modalities, which provide trainees with a risk-free environment to develop procedural skills through repetition, feedback, and graduated difficulty.^3^

XR encompasses a range of simulation platforms that include entirely digital environments (VR), overlays of digital information onto the physical world (AR), or hybrid environments that combine physical and virtual interaction (mixed reality).^2^^,^^17^^,^^20^ In orthopedic surgery, early evidence suggests that XR platforms can shorten the learning curve for complex arthroplasty tasks. A systematic review by Pettinelli et al^23^ found that VR training was associated with a small standardized improvement in procedure time compared with controls,^18^ and a 2025 study by Dey Hazra et al^5^ showed that AR-assisted intraoperative navigation helped junior surgeons improve their glenoid component placement accuracy in cadavers. In addition, XR simulators offer superior standardization and performance metrics,^15^ and XR training has been shown to improve operative performance in surgical training.^15^^,^^28^,^30^

Despite growing interest among surgical trainees,^16^^,^^24^ the specific impact of XR training on shoulder arthroplasty is poorly understood. XR training aligns well with modern surgical training principles, including deliberate practice, objective feedback, and cognitive task decomposition.^8^ Unlike passive observation or single-pass cadaver lab sessions, XR allows for iterative engagement with core tasks and is less resource-intensive.^11^^,^^21^ This may democratize surgical skills acquisition, reducing variability in residency and fellowship training programs.

Despite promising early data, variability in XR platforms and study design has made it difficult to generalize results. Our study seeks to address this by analyzing the effects of XR-based training on 2 distinct but complementary domains. First, XR simulation may accelerate learning and improve operative efficiency and technical proficiency during training. Second, XR may be used for procedural guidance, providing intraoperative visualization and navigation support to improve the accuracy of glenoid guidewire placement.

Accordingly, we performed 2 meta-analyses addressing (1) the effect of XR-based education on procedure time and objective performance scoring and (2) the effect of XR guidance on absolute error in glenoid guidewire version, inclination, and entry point relative to the freehand technique.

## Methods

This meta-analysis was conducted in accordance with the Preferred Reporting Items for Systematic Reviews and Meta-Analyses guidelines.^22^ Sources were obtained from PubMed, Embase, and Scopus using a custom search strategy. A PubMed search strategy was constructed using terms related to Virtual Reality, Augmented Reality, Mixed Reality, Extended Reality, Shoulder, Scapula, Glenoid, and Arthroplasty. The format was then adjusted to fit Embase and Scopus. All 6 search strategies can be found in the Appendix. For both analyses, 1 author (M.R.) performed article screening and sent prospective articles to another author (E.C.) for review and approval.

Inclusion in the XR education meta-analysis was limited to randomized controlled trials or prospective studies that involved the comparison of XR training to standard training (didactic instruction, bench-top models, or cadaveric training). Eligible studies were required to use quantitative outcome measures and had to include information regarding the specific XR system utilized by the experimental group, conventional training utilized by the control group, and procedure used for final evaluation. Database searches were performed on May 16, 2025, and 24 studies were identified. After applying criteria, 3 studies involving 55 participants were included. Inclusion in the XR glenoid guidewire meta-analysis was limited to quasi-experimental studies comparing XR-assisted glenoid guidewire placement to freehand guidewire placement. Database searches were performed on May 21, 2025, and 35 studies were identified. After applying criteria, 7 studies involving 349 guidewire placements were included.

Data extraction was performed by 1 author (M.R.), and potentially eligible articles were reviewed with a second author (E.C.) before final inclusion. Data extraction was performed by 1 author (M.R.) using a standardized Excel spreadsheet. Extracted variables included study design, sample size, XR system, XR modality/type, evaluated procedure or task, comparator training/guidance method, and outcome data (means and standard deviations). Because extracted data were not independently duplicated by a second reviewer, the possibility of extraction bias cannot be fully excluded.

Table I outlines the 3 studies included in the education meta-analysis. All 3 studies used OSAT (Objective Structured Assessment of Technical Skills) scores to evaluate operative proficiency and recorded the time it took each participant to complete their task. Table II outlines the 7 studies included in the glenoid guidewire meta-analysis. For the glenoid guidewire analysis, absolute version and inclination error were extracted as reported by each original study. In general, error represented the absolute deviation between the achieved guidewire trajectory and the planned or target trajectory defined by the investigators, commonly using pre-operative computed tomography-based planning or platform-specific target axes. Because the exact reference standard was not identical across studies, differences in the definition of the target trajectory may have contributed to between-study heterogeneity.

**Table I:** Characteristics of studies included in the XR education meta-analysis.

Study authors	Study design	n	XR platform	Evaluated procedure	Outcome measures
Lohre et al^18^	RCT	26	PrecisionOS	Cadaveric glenoid exposure	OSAT scores and time to completion
Lohre et al^19^	RCT	24	PrecisionOS platform 3.0	Cadaveric reverse shoulder arthroplasty	OSAT scores and time to completion
Crockatt et al^4^	RCT	20	PrecisionOS	Cadaveric glenoid baseplate implantation	OSAT scores and time to completion

*XR*, extended reality; *RCT*, randomized control trial.

Summary of included XR education randomized controlled trials, including study design, sample size, XR platform, evaluated procedure/task, and primary outcome measures (OSAT score and time to completion).

**Table II:** Characteristics of studies included in the glenoid guidewire meta-analysis.

Study authors	Setting	n (shoulders; surgeons)	XR system	XR type	Outcome measures
Dordain et al^6^	Cadaveric	16 shoulders; 2 surgeons	Pixee Medical AR-HMD shoulder guidance	AR navigation (head-mounted display)	Version error, inclination error, and entry point error
Erickson et al^7^	Benchtop (B2 sawbones)	60 shoulders; 5 surgeons	Microsoft HoloLens 2 (custom Unity holographic guidance)	MR overlay (non-navigated)	Version error, inclination error, and entry point error
Fleet et al^9^	Benchtop (3D-printed arthritic glenoids)	60 shoulders; 3 surgeons	Stryker Blueprint Mixed Reality Guidance + Microsoft HoloLens 2 (MR-NAV)	MR navigation (Holoblueprint)	Version error, inclination error, and entry point error
Frantz et al^10^	Phantom/benchtop (Sawbones + skin envelope)	18 shoulders; 2 surgeons	Microsoft HoloLens 2 (custom AR app; inside-out IR tool tracking)	AR navigation (tracked)	Version error, inclination error, and entry point error
Imbert et al^12^	Benchtop (3D-printed arthritic glenoids)	60 shoulders; 2 surgeons	Stryker Blueprint Mixed Reality Guidance + Microsoft HoloLens 2	MR guidance (non-nav overlay)	Version error, inclination error, and entry point error
Italia et al^13^	Benchtop (3D-printed scapulae)	60 shoulders; 3 surgeons	Microsoft HoloLens 2 (Akunah overlay app)	MR overlay (manual hologram alignment)	Version error, inclination error, and entry point error
Kopriva et al^14^	Clinical (post-operative CT)	97 shoulders; 1 surgeon	Stryker Blueprint Mixed Reality + Microsoft HoloLens	MR overlay (intraoperative visualization)	Version error, inclination error, and entry point error

*VR*, virtual reality, *AR*, augmented reality, *MR*, mixed reality; *XR*, extended reality; 3D, 3-dimensional; CT, computed tomography.

Summary of included quasi-experimental studies comparing XR-assisted glenoid guidewire placement to freehand technique, including study setting (benchtop/cadaveric/clinical), number of specimens and surgeons, XR system and modality/type, and reported outcomes (absolute version error, inclination error, and entry point error).

Due to clinical and methodological heterogeneity, both analyses were conducted using inverse-variance random-effects models (DerSimonian–Laird). Heterogeneity was assessed using I^2^ and τ^2^. Statistical analysis and figure generation were performed in Python 3.12.7 (Anaconda distribution).

## Results

**Figure 1:**
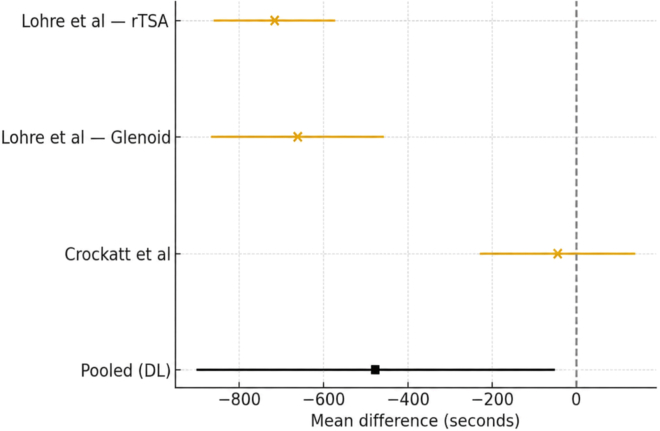
XR education meta-analysis: time to completion. Forest plot comparing time to completion (seconds) for XR-trained participants versus standard training controls across included XR education studies. Effect sizes are presented as mean differences (XR − control) with 95% confidence intervals (CIs) using a random-effects (DerSimonian–Laird) inverse-variance model. Negative values favor XR (shorter completion time). *DL*, DerSimonian-Laird random-effects pooling method; *rTSA*, reverse total shoulder arthroplasty; *XR*, extended reality.

**Figure 2:**
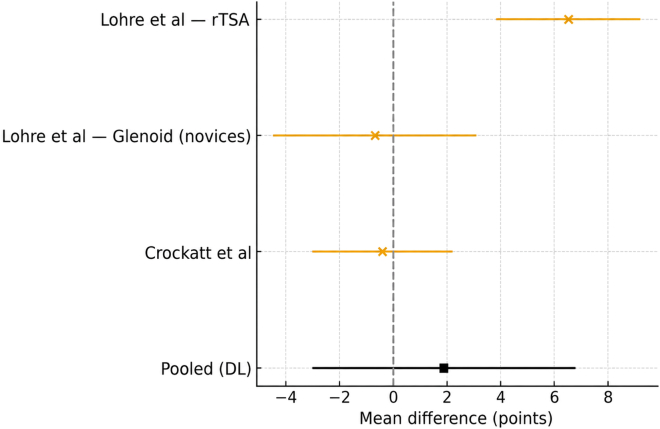
XR education meta-analysis: OSAT scores. Forest plot comparing Objective Structured Assessment of Technical Skill (OSAT) scores for XR-trained participants versus standard training controls across included XR education studies. Effect sizes are presented as mean differences (XR − control) with 95% CIs using a random-effects (DerSimonian–Laird) inverse-variance model. Positive values favor XR (higher OSAT scores). *CIs*, confidence intervals; *DL*, DerSimonian-Laird random-effects pooling method; *rTSA*, reverse total shoulder arthroplasty; *XR*, extended reality.

Across the 3 XR education studies, XR-trained participants completed procedures an average of 477 seconds faster than controls (95% confidence interval [CI]: −902 to −53; I^2^ = 94.3%, τ^2^ = 132,415). Time-to-completion data are listed in Table III, and the forest plot is shown in Figure 1. Although the pooled estimate favored XR training, the direction and magnitude of benefit were not uniform across all 3 studies. Both studies by Lohre et al^18^^,^^19^ demonstrated clear reductions in task completion time with XR training, whereas Crockatt et al^4^ showed an insignificant difference, contributing to the high observed heterogeneity. Unlike time-to-completion, OSAT scores did not significantly differ between XR and control groups (pooled mean difference: 1.87; 95% CI: −2.99 to 6.73; I^2^ = 87.7%, τ^2^ = 16.08). OSAT values by study are provided in Table IV, and the forest plot is shown in Figure 2.

**Table III:** XR education study outcome data: time to completion.

Study	Group	n	Mean time (s)	SD
Lohre et al^18^	Novice control	11	1,200	240
	Novice XR	11	660	180
	Expert control	3	1,140	300
	Expert XR	4	240	100
Lohre et al^19^	Control	15	966	156
	XR	15	246	150
Crockatt et al^4^	Cadaver-trained	11	591	192
	XR	7	546	158

*SD*, standard deviation; *XR*, extended reality.

Study-level time-to-completion data (seconds) for XR and control groups, including group definitions (eg, novice/expert where applicable), sample sizes, means, and standard deviations.

**Table IV:** XR education study outcome data: OSAT scores.

Study	Group	n	Score	SD
Lohre et al^18^	Novice control	11	18.5	4.11
	Novice XR	11	17.11	2.5
Lohre et al^19^	Control	15	15.4	3.2
	XR	15	21.15	2.5
Crockatt et al^4^	Cadaver-trained	11	24.65	1.26
	XR	7	24.30	3.4

*SD*, standard deviation; *XR*, extended reality.

Study-level OSAT score data for XR and control groups, including group definitions, sample sizes, means, and standard deviations. OSAT values reflect the scoring approach used in each included trial.

**Figure 3:**
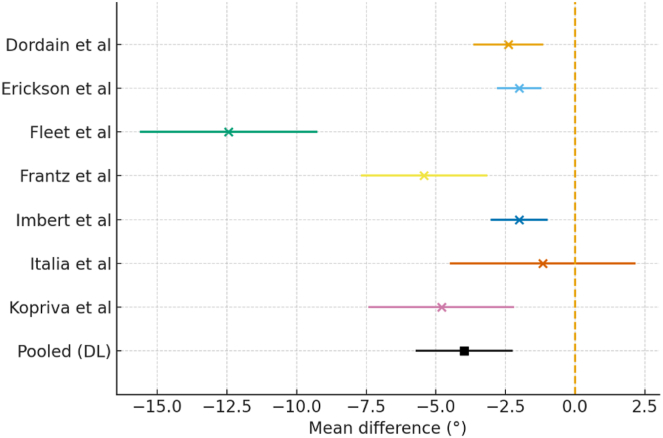
Glenoid guidewire meta-analysis: version error. Forest plot comparing absolute version error (degrees) for XR-assisted glenoid guidewire placement versus freehand placement across included guidewire studies. Effect sizes are presented as mean differences (XR − freehand) with 95% CIs using a random-effects (DerSimonian–Laird) inverse-variance model. Negative values favor XR (lower version error). *CIs*, confidence intervals; *DL*, DerSimonian-Laird random-effects pooling method; *XR*, extended reality.

**Figure 4:**
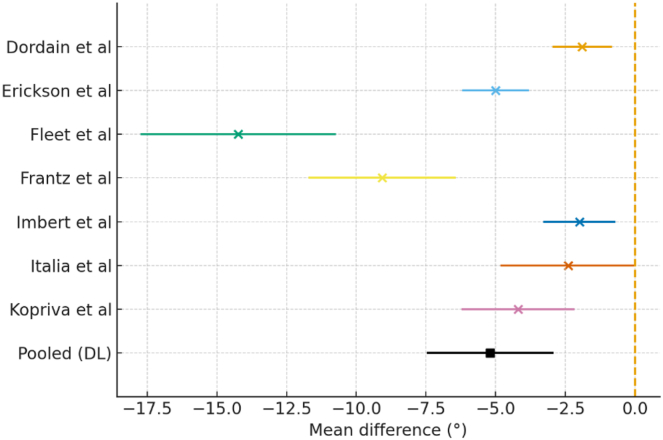
Glenoid guidewire meta-analysis: inclination error. Forest plot comparing absolute inclination error (degrees) for XR-assisted glenoid guidewire placement versus freehand placement across included guidewire studies. Effect sizes are presented as mean differences (XR − freehand) with 95% CIs using a random-effects (DerSimonian–Laird) inverse-variance model. Negative values favor XR (lower inclination error). *CIs*, confidence intervals; *DL*, DerSimonian-Laird random-effects pooling method; *XR*, extended reality.

**Figure 5:**
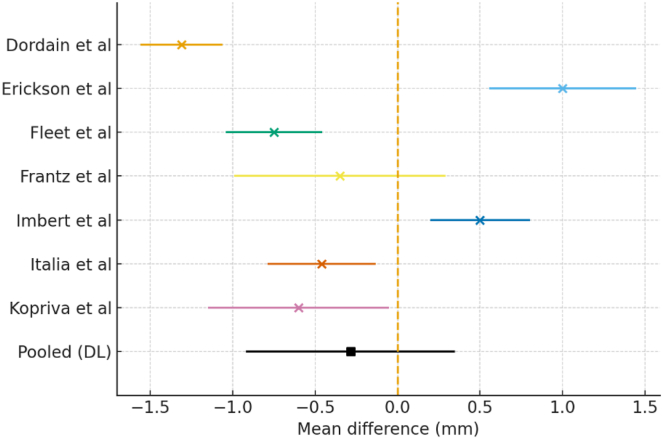
Glenoid guidewire meta-analysis: entry point error. Forest plot comparing entry point error (mm) for XR-assisted glenoid guidewire placement versus freehand placement across included guidewire studies. Effect sizes are presented as mean differences (XR − freehand) with 95% CIs using a random-effects (DerSimonian–Laird) inverse-variance model. Negative values favor XR (lower entry point error). *CIs*, confidence intervals; *DL*, DerSimonian-Laird random-effects pooling method; *XR*, extended reality.

Across the 7 glenoid guidewire studies, XR guidance reduced version error by an average of 4° compared to freehand (95% CI: −5.8 to −2.3; I^2^ = 86.2%, τ^2^ = 4.94) (Fig. 3). XR guidance also reduced inclination error by an average of 5.2° compared to freehand (95% CI: −8.4 to −3.0; I^2^ = 91.1%, τ^2^ = 9.23) (Fig. 4). Entry point error was not significantly different between XR-guided procedures and freehand (pooled mean difference: −0.29 mm; 95% CI: −0.94 to 0.36; I^2^ = 93.4%, τ^2^ = 0.71) (Fig. 5). Glenoid guidewire error data are listed in Table V.

**Table V:** Glenoid guidewire outcome data: absolute error measures.

Study	Group	n (specimens)	Version error (degrees)	SD	Inclination error (degrees)	SD	Entry point error (mm)	SD
Dordain et al^6^	Freehand	16	5.5	4.4	5.1	3.8	1.1	0.85
	XR	16	0.8	0.5	0.15	1.7	0.5	0.15
Erickson et al^7^	Freehand	30	19.52	15.27	16.56	8.37	2.27	0.95
	XR	30	18.36	15.25	11.22	5.69	1.75	0.84
Fleet et al^9^	Freehand	26	3	2.7	4	3.1	1.5	0.75
	XR	26	1	1.4	2	0.5	2	0.56
Frantz et al^10^	Freehand	7	7.00	2.59	15.67	3.4	1.41	0.47
	XR	7	0.58	1.5	0.60	1.24	1.7	0.64
Imbert et al^12^	Freehand	45	20.33	17.39	22.33	18.62	2.20	0.15
	XR	45	1.89	1.4	2.15	1.8	1.39	1.4
Italia et al^13^	Freehand	60	7.0	3.0	18.0	4.0	2.3	1.1
	XR	60	4.0	3.0	8.0	5.0	3.3	2.0
Kopriva et al^14^	Freehand	72	4.8	3.15	4.3	3.4	3.1	0.79
	XR	31	2.3	2.1	2.4	1.8	1.79	0.41

*SD*, standard deviation; *XR*, extended reality.

Study-level absolute error data for XR-assisted versus freehand glenoid guidewire placement, including sample sizes, means, and standard deviations for version error, inclination error, and entry point error. Absolute error reflects deviation from the target or planned guidewire position as defined by each original study, which varied by study design and planning platform.

## Discussion

This article synthesizes the current evidence on XR in shoulder arthroplasty across 2 domains: (1) XR-based surgical education and (2) XR-assisted guidance for glenoid guidewire placement. The primary findings indicate that XR education is associated with improvements in operative efficiency (approximately 8 minutes faster task completion than controls), while technical proficiency (measured by OSAT scores) is not significantly affected. Furthermore, XR guidance demonstrated significant reductions in angular error for both version and inclination during guidewire placement, while entry point error did not show a significant difference.

The clinical relevance of these effect sizes requires cautious interpretation. A pooled reduction of approximately 4° in version error and 5.2° in inclination error suggests that XR guidance improves angular accuracy, but the degree to which these differences influence implant survivorship, complications, or functional outcomes is uncertain. Similarly, a 477-s improvement in simulated task completion time may be meaningful in the context of short cadaveric or simulated procedures, but it should not be assumed to directly translate into shorter operative time. No established minimal clinically important difference exists for guidewire version error, inclination error, entry point error, OSAT score, or simulated completion time in this specific context. Therefore, these findings should be interpreted as improvements in technical and educational surrogate outcomes rather than definitive evidence of improved clinical outcomes.

A particularly important unanswered question is whether XR guidance reduces clinically important outliers in implant or guidewire positioning. Mean reductions in angular error may underestimate or overestimate clinical value if they do not capture the frequency of extreme malpositioning. In the included studies, outlier rates were not consistently reported in a manner that allowed pooled analysis. This limits the ability of the present meta-analysis to determine whether XR prevents the most consequential positioning errors. Future XR studies should report not only mean version, inclination, and entry point error but also the proportion of cases exceeding predefined error thresholds, such as deviations greater than 5°, 10°, or other clinically justified cutoffs. These outlier-based outcomes may be more relevant to patient safety and implant longevity than small improvements in average error alone.

Time-to-completion reductions may reflect the benefits of repeated exposure, cognitive rehearsal, and task decomposition that XR platforms enable. Unlike cadaveric training, XR can offer rapid repetition with immediate feedback and may reduce extraneous cognitive load during the evaluated procedure. This could plausibly translate into faster execution even when global performance scores do not clearly separate groups. For intraoperative guidance, XR systems may offer the greatest advantage in maintaining a surgeon's intended trajectory (version and inclination), especially when holographic overlays reinforce alignment. Entry point error, however, may be more sensitive to initial landmark identification and soft-tissue constraints. These factors could plausibly blunt improvements in the entry point even when trajectory control improves. The lack of significant entry point differences supports the possibility that XR systems may be better at improving angular guidance than start-point localization.

Taken together, these findings support XR as a promising adjunct across the field of shoulder arthroplasty. For trainees, XR education may provide scalable opportunities to rehearse tasks and improve procedural skill. For surgeons performing arthroplasty on real patients, XR guidance may enhance angular accuracy of guidewire placement—an important determinant of component alignment.

The relevance of these findings is amplified by the unique anatomical and biomechanical challenges inherent to shoulder arthroplasty. Proper glenoid component positioning remains a critical determinant of long-term implant survival and functional outcomes, and malpositioning can lead to early loosening, scapular notching, instability, and acromial or scapular spine stress fractures.^27^ Traditional learning environments such as cadaveric labs and operating room experience are limited by cost, variability in pathology, and operative time constraints. In contrast, XR training platforms offer reproducible, high-volume exposure to critical steps of the operation.

While the results of our meta-analysis may be promising, additional research is needed. In addition, the value of XR implementation must also be weighed against cost and setup time. Despite the potential cost benefits outlined previously, XR systems require significant capital investment, software licensing, and maintenance. These drawbacks may be easier to justify if XR reduces major malpositioning outliers, improves training efficiency, or benefits complex anatomy, but the current evidence base does not yet define the threshold at which these benefits outweigh implementation costs. Future studies should include cost-effectiveness analyses and report setup time, registration time, and technical burden. We believe that future studies would also benefit from standardized shoulder arthroplasty performance metrics, multicenter, randomized controlled design, and longer retention and operating room transfer assessments. Such data will clarify where XR sits within orthopedic surgery curricula and whether it can ultimately drive patient-level benefits.

## Limitations

The limitations of our meta-analysis may temper its applicability. The education meta-analysis includes only 3 randomized controlled trials (55 participants), limiting power to detect differences in performance scoring and limiting subgroup analyses (eg, novice vs. expert; platform differences). In addition, the XR guidance evidence base includes quasi-experimental studies across heterogeneous settings (benchtop, cadaveric, and clinical), with variability in XR systems and measured protocols. Data extraction was performed by a single reviewer using a standardized spreadsheet. Although prospective articles were reviewed with a second author before final inclusion, extracted numerical data were not independently duplicated by a second reviewer. This introduces a potential risk of extraction error or reviewer bias.

Regarding our results, pooled analyses demonstrated substantial heterogeneity, with I^2^ values ranging from 86% to 94% across primary outcomes. This heterogeneity likely reflects differences in XR platforms, study setting, comparator technique, specimen type, and definitions of target guidewire position. Random-effects modeling was selected because true effect sizes were expected to vary across these clinically and methodologically diverse studies. However, random-effects modeling does not eliminate the underlying heterogeneity, and the pooled estimates should therefore be interpreted as average effects across heterogeneous experimental settings rather than precise predictions of benefit. Most studies focus on short-term or immediate outcomes rather than long-term clinical endpoints such as implant survivorship, complications, or durability of skill transfer to the operating room. In addition, the reference standard used to calculate version and inclination error was not fully standardized across included guidewire studies. Some studies used pre-operative planning targets or platform-specific planning software, while others reported deviation from study-defined target trajectories. This variation may have contributed to heterogeneity and limits direct comparison across studies. Finally, the cost of capital investment and maintenance, logistic requirements, and institutional acceptance were not reported. These are all factors that influence real-world adoption of XR training, and further studies are warranted to fully determine how they will impact the practicality of XR implementation.

## Conclusion

XR-based tools appear to improve selected educational and technical outcomes in shoulder arthroplasty, but their impact may be domain-specific and should be interpreted cautiously. XR education was associated with faster simulated task completion, while XR guidance was associated with improved angular accuracy of glenoid guidewire placement compared with the freehand technique. Entry point error did not significantly differ between XR-guided and freehand approaches. Because minimal clinically important difference values, outlier reduction, cost-effectiveness, and patient-level outcomes remain insufficiently studied, future research should determine whether these improvements in surrogate measures translate into clinically meaningful benefits.

## Disclaimers:

Funding: No funding was disclosed by the authors.

Conflicts of interest: The authors, their immediate families, and any research foundations with which they are affiliated have not received any financial payments or other benefits from any commercial entity related to the subject of this article.
