# Supplementary material for: Utility of extended reality in shoulder arthroplasty: a meta-analysis of training efficiency and glenoid guidewire accuracy
Source: J Shoulder Elb Arthroplast. 2026 Jun 24;10(3):100051. doi: 10.1016/j.jsea.2026.100051 (PMC13400657; doi:10.1016/j.jsea.2026.100051)
Supplement: Supplementary Appendix S1 [file mmc1.docx]

**Appendix**

XR Education/Training Query:

("virtual reality" OR “augmented reality” OR “mixed reality” OR “extended reality”) AND (curriculum[mesh] OR curriculum[tw] OR "Internship and Residency"[mesh] OR "Schools, Medical"[mesh] OR "education, medical"[mesh] OR education[sh] OR training*[tw] OR "Competency-Based Education "[mesh]) AND ("Orthopedic Procedures"[mesh] OR Orthopedics[mesh] OR ((surgery*[tw] OR surgical[tw] OR surgeon*[tw] OR surgery[sh]) AND (orthopedic* OR orthopaedic*))) AND (“shoulder” OR “scapula” OR “glenoid”) AND (“arthroplasty”) NOT (review[pt] NOT ("systematic review"[pt] OR "meta analysis"[pt] OR "systematic review"[ti] OR "meta analysis"[ti] OR metaanalysis[ti] OR "scoping review"[ti])) NOT (rehabilitation*[ti] OR "exercise therapy"[mesh])

XR Glenoid Guidewire Query:

("shoulder arthroplasty" OR "reverse shoulder arthroplasty" OR RSA OR "total shoulder arthroplasty" OR TSA) AND (glenoid OR guidewire OR "guide wire" OR pin OR "K-wire" OR "Kirschner wire" OR "central guide" OR "baseplate guide") AND ("extended reality" OR XR OR "mixed reality" OR MR OR "augmented reality" OR AR OR "virtual reality" OR VR OR hologram* OR "head-mounted display" OR HMD OR HoloLens) AND (navigat* OR guid* OR "image-guided" OR "computer-assisted" OR "computer assisted" OR overlay OR "surgical planning" OR "patient-specific" OR "patient specific")
